# Systemic Treatment with Fas-Blocking Peptide Attenuates Apoptosis in Brain Ischemia

**DOI:** 10.3390/ijms25010661

**Published:** 2024-01-04

**Authors:** Sungeun Chung, Yujong Yi, Irfan Ullah, Kunho Chung, Seongjun Park, Jaeyeoung Lim, Chaeyeon Kim, Seon-Hong Pyun, Minkyung Kim, Dokyoung Kim, Minhyung Lee, Taiyoun Rhim, Sang-Kyung Lee

**Affiliations:** 1 Department of Bioengineering and Institute of Nanoscience and Technology, Hanyang University, Seoul 04763, Republic of Koreayiyujong@gmail.com (Y.Y.); minhyung@hanyang.ac.kr (M.L.); 2 Department of Internal Medicine, Yale University, New Haven, CT 06520, USA; 3 Lerner Research Institute, Cleveland Clinic, Cleveland, OH 44195, USA; 4 Department of Biomedical Science, Graduate School, Kyung Hee University, Seoul 02447, Republic of Korea; dkim@khu.ac.kr

**Keywords:** systemic treatment, Fas-blocking peptide, apoptosis, brain ischemia, neuronal injury, Fas-mediated cell death, ischemic strokes, Fas-signaling, neuronal cell death, blood–brain barrier

## Abstract

Apoptosis plays a crucial role in neuronal injury, with substantial evidence implicating Fas-mediated cell death as a key factor in ischemic strokes. To address this, inhibition of Fas-signaling has emerged as a promising strategy in preventing neuronal cell death and alleviating brain ischemia. However, the challenge of overcoming the blood–brain barrier (BBB) hampers the effective delivery of therapeutic drugs to the central nervous system (CNS). In this study, we employed a 30 amino acid-long leptin peptide to facilitate BBB penetration. By conjugating the leptin peptide with a Fas-blocking peptide (FBP) using polyethylene glycol (PEG), we achieved specific accumulation in the Fas-expressing infarction region of the brain following systemic administration. Notably, administration in leptin receptor-deficient db/db mice demonstrated that leptin facilitated the delivery of FBP peptide. We found that the systemic administration of leptin-PEG-FBP effectively inhibited Fas-mediated apoptosis in the ischemic region, resulting in a significant reduction of neuronal cell death, decreased infarct volumes, and accelerated recovery. Importantly, neither leptin nor PEG-FBP influenced apoptotic signaling in brain ischemia. Here, we demonstrate that the systemic delivery of leptin-PEG-FBP presents a promising and viable strategy for treating cerebral ischemic stroke. Our approach not only highlights the therapeutic potential but also emphasizes the importance of overcoming BBB challenges to advance treatments for neurological disorders.

## 1. Introduction

Ischemic stroke occurs due to a sudden restriction or blockage of the blood flow to the brain [1]. As a result, the affected part of the brain is unable to receive oxygen and nutrients, leading to damage or death of brain cells [1,2,3]. The two main subtypes of ischemic stroke are thrombotic strokes, which occur when a blood clot forms in an artery supplying blood to the brain, and embolic strokes, which occur when a blood clot or debris from elsewhere in the body travels to the brain through the blood stream [3,4]. Ischemic strokes constitute approximately 80% of global stroke cases, presenting a severe and life-threatening medical emergency [5].

The primary treatment guideline for acute ischemic stroke involves a type of thrombolytic therapy that involves the intravenous administration of a recombinant tissue-type plasminogen activator (tPA) within 3 to 4.5 h of a stroke [6]. However, this approach is constrained by a narrow time window for effectiveness, leading to only 3.4–5.2% of eligible stroke patients receiving tPA therapy [7]. In order to augment the effectiveness of tPA, some studies have explored combination therapies with other interventions, both drug and nondrug [8]. Apart from thrombolytic therapy, there is currently a lack of effective methods in treating ischemic stroke [9]. In fact, between 1995 and 2015, 430 drug candidates for ischemic stroke were identified, but only 19 (4%) successfully entered the market [10]. 

The complex interplay of molecular mechanisms in ischemic stroke initiates a series of events that impact neural substrates [11]. Fas-mediated apoptosis can impact neural circuits in the central nervous system, particularly in the brain [12,13]. Fas (CD95) is a cell surface receptor that plays a role in apoptosis, or programmed cell death, triggering a signaling pathway leading to apoptosis when it binds to its ligand (FasL) [12,14,15]. The impact of Fas-mediated apoptosis on neural circuits varies depending on the specific region of the brain and the type of cells that are involved [13,16,17,18]. Animal models, including the middle cerebral artery occlusion (MCAO) model and the photothrombotic model [19,20,21,22,23], have revealed an upregulation of Fas and FasL expression, implicating the Fas–FasL signaling system in the pathogenesis of stroke [15,24,25]. Interventions targeting the Fas–FasL axis have demonstrated noteworthy outcomes under conditions of cerebral ischemia. The pharmacological elimination of FasL or genetic suppression of the Fas–FasL system has been observed to exert a neuroprotective effect [14,26,27,28], highlighting the role of the Fas–FasL signaling cascade in the events leading to stroke-induced neuronal damage [29,30]. The identification of the Fas–FasL system as a key player in ischemic stroke pathogenesis has led to the exploration of innovative therapeutic strategies [9,31]. One avenue of research is the inhibition of apoptosis in the penumbra, a region of the brain that is involved in the initiation of a gradual process of apoptosis around the ischemic core, induced by reduced blood flow from brain ischemia [9,32]. The inhibition of the Fas–FasL interaction holds potential not only for novel and targeted treatment approaches for ischemic stroke injuries, but also for various central nervous system (CNS) diseases, including epilepsy, multiple sclerosis, Parkinson’s disease, Huntington’s disease, Alzheimer’s disease, and stroke [27,33,34,35,36,37,38].

A significant obstacle to pharmaceutical stroke treatments, however, is the presence of the blood–brain barrier (BBB), which limits drug access to the central nervous system (CNS) [39,40]. Only small, lipid-soluble molecules with a molecular weight < 400 Da can passively diffuse through the BBB via the transcellular lipophilic pathway without expending metabolic energy [41]. To overcome this obstacle, various approaches for successful drug delivery across the BBB have been explored, including carrier-mediated transport, adsorptive-mediated transport, and receptor-mediated transcytosis [42,43,44]. One prior study employed the RVG9R peptide as a carrier for delivering therapeutic siRNA to the brain, targeting the nicotinic acetylcholine receptor [45,46]. Receptor-mediated delivery approaches include transferrin, insulin, low-density lipoprotein, glutamate and the leptin receptor [47]. Among these strategies, leptin has shown particular promise due to the prevalent expression of the leptin receptor in the brain region [48,49]. Liu et al. demonstrated successful fluorescent expression in the brain through the systemic delivery of a leptin-derived peptide conjugated with PEGylated dendrigraft nanoparticles, complexed with fluorescence-labelled DNA [48]. This method holds clinical relevance, offering advantages such as conventional systemic administration, potential for multiple dosing with reduced risk of BBB restriction, cost-effectiveness, and the potential for use without clinical supervision [50]. Building upon insight from prior studies, the primary objective of this study is to evaluate the therapeutic potential of systemically delivering a Fas-blocking peptide (FBP) to inhibit the Fas–FasL interaction, thereby reducing neuronal cell death in ischemic stroke [48,51]. 

## 2. Results

### 2.1. The Leptin-PEG-FBP Conjugate Inhibits Apoptosis in Hypoxic-N2a Cells

**Figure 1:**
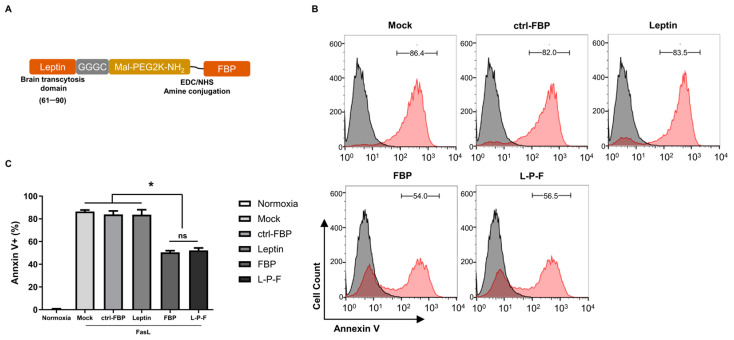
The inhibitory effect of FBP or leptin-PEG-FBP (L-P-F) on FasL-induced apoptosis in hypoxic-N2a. (**A**) Schematic illustration of the L-P-F peptide synthesis. (**B**,**C**) The N2a cells were cultured in a hypoxic condition in the presence of membrane-bound soluble FasL (5 nM) for 6 h, as described in the Methods section. The cells were then treated with 300 μM of the indicated peptides for 6 h and examined for Annexin V-PE staining. (**B**) Representative histograms illustrate the percentage of apoptosis with the peptide treatments, depicting live cells (in black) and apoptotic cells (in red). (**C**) Bar graph represents the percentage of Annexin V positive cells with the peptide treatments. Normoxia describes cells cultured in normal oxygen conditions. Hypoxia describes cells cultured in hypoxic conditions. The bar graph indicates the average ± SD of three independent experiments. The data in (**C**) was analyzed using Mann–Whitney U test (* *p* < 0.05; ns: not-significant).

In a prior study, we observed that the Fas-blocking peptide (FBP) effectively disrupted Fas–FasL interactions, thereby inhibiting Fas-mediated apoptosis both in vitro and in vivo when delivered intranasally [30]. In this study, we explored the impact of FBP through the clinically relevant systemic route of delivery. To conjugate FBP with leptin, a brain-targeting peptide that effectively penetrates the BBB, we employed polyethylene glycol (PEG) as a linker. We initially evaluated the toxicity of PEG of various sizes in Jurkat cells. The treatment of Jurkat cells with membrane-bound FasL induced approximately 40% apoptosis compared to non-FasL (normal) cells. Both FBP alone and FBP conjugated with PEG-1K, and PEG-2K significantly reduced FasL-mediated apoptosis, demonstrating that the presence of PEG did not interfere with the FBP’s ability to inhibit the Fas–FasL interaction. However, PEG-3.5K, PEG-5K, and PEG-10K showed significantly higher levels of apoptosis, indicating PEG-associated toxicity when used beyond PEG-2K (Figure S1). Our results align with other studies that suggest that larger sizes of PEG induce toxicity in cells [52]. Based on our findings, we conjugated PEG-2K to FBP (PEG-FBP) and then linked it with the leptin peptide. The leptin peptide demonstrated successful delivery to the brain compared to the negative control peptide RV-MAT [45] (Figure S2). The conjugation schematic is illustrated in Figure 1A. 

To evaluate the effect of leptin-PEG-FBP on Fas-mediated apoptosis, hypoxic Neuro2a mouse neuroblastoma (N2a) cells were treated with FasL (0.1 mM), followed by a treatment with the peptides indicated in Figure 1B. Our results showed that both FBP and leptin-PEG-FBP significantly reduced cell death upon exposure to FasL, as indicated by the 40% decrease in Annexin V positivity (Figure 1B). In contrast, the control FBP (ctrl-FBP) and the leptin alone did not demonstrate a reduction in Fas-mediated apoptosis compared to that of the control. Interestingly, leptin-PEG-FBP did not lose its protective effect and behaved similarly to FBP alone in cultured cells (Figure 1C). Our findings suggest that leptin-PEG-FBP maintains the anti-apoptotic effect of FBP.

### 2.2. Intravenous Delivery of Leptin-PEG-FBP Localizes to Fas-Expressing Brain Regions in Photothrombotic Stroke Mouse Models of Ischemia

**Figure 2:**
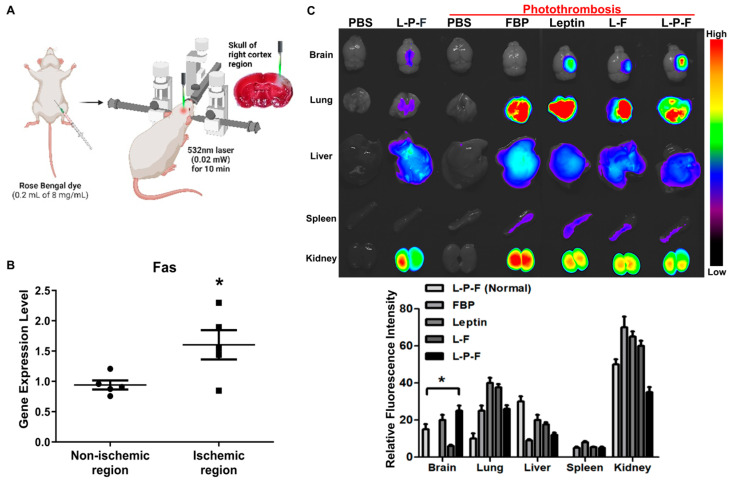
Systemically delivered leptin-PEG-FBP (L-P-F) localizes to Fas-expressing brain regions in the photothrombotic mouse model of ischemia. (**A**) Schematic illustration of the photothrombotic stroke model. (**B**) mRNA quantification of Fas expression in non-ischemic and ischemic region of photothrombotic stroke mouse brain, normalized to GAPDH. Data represent the average ± SD of three independent experiments from *n* = 5. (**C**) The representative images and quantification of fluorescence intensity in indicated organs after necropsy. Alexa^647^-labelled peptides were systemically delivered (i.v.) to control and ischemic mice, and organs were collected at 12 h post-injection, imaged on Kodak imaging station. Control mice were inoculated with PBS or leptin-PEG-FBP. Fluorescence was labeled on FBP in both leptin-FBP (L-F) and in leptin-PEG-FBP (L-P-F). Fluorescence intensities, measured in arbitrary pixel values, were normalized to intensities in the PBS-treated group. Data represent mean ± SD (*n* = 5 per group). The data in (**B**) and (**C**, **bottom**) were analyzed using Mann–Whitney U test (* *p* < 0.05).

Clinical evidence indicates that the Fas–FasL apoptotic signal plays a key role in cell death during ischemic strokes [27]. Previous research has shown that Fas mRNA and protein levels increase in rat brains a few hours after ischemic stroke [15,24]. In our investigation using a mouse model of brain ischemia induced using photothrombosis (Figure 2A), we first assessed the level of Fas expression in the ischemic region. We observed a significant increase in the level of Fas mRNA expression in the ischemic region (right hemisphere) compared to the non-ischemic region (left hemisphere) (Figure 2B). Subsequently, we evaluated the brain delivery of Alexa^647^-labeled peptides through systemic delivery (i.v.) in both wildtype and ischemic stroke mouse models. Organ imaging conducted 12 h post-single administration of 2.5 mg/kg of peptides demonstrated a pronounced localization of the leptin-PEG-FBP peptide, followed by leptin alone and leptin-FBP (L-F) in the ischemic region of the brain (Figure 2C). In addition, compared to the ischemic region (right hemisphere), negligible fluorescence was detected in the non-ischemic region (left hemisphere). This observation aligns with our expectations, as only the right hemisphere undergoes apoptosis following the photothrombotic procedure (Figure 2C). However, in the absence of the leptin peptide (Alexa^647^-FBP), we could not detect Alexa^647^ signals in the infarcted regions of ischemic brains, suggesting that the FBP peptide alone is unable to get through the blood–brain barrier (BBB). 

Similarly, the administration of peptides in wildtype mice (non-ischemic) did not reveal the presence of Alexa^647^, underscoring the crucial role of leptin-mediated peptide delivery. The low intensity of Alexa^647^ signals observed in leptin-PEG-FBP-administered control mice indicate the specific delivery of FBP to Fas-expressing ischemic regions of the brain mediated by the leptin/leptin receptor interaction. In addition, we observed the localization of Alexa^647^ signals in almost all periphery organs including the lung, liver, and kidney. This observation can be attributed to the metabolic processes associated with drugs administrated intravenously [53,54].

Our data indicate that FBP localization in the ischemic regions of brain is caused by the binding of FBP to Fas-expressing neuronal cells. Furthermore, leptin-PEG-FBP’s strong localization at the infarcted region compared to that of leptin-FBP highlights the importance of PEG in facilitating the transport of the leptin-PEG-FBP peptide through the cerebrospinal fluid (CSF). 

### 2.3. Leptin-Mediated Transcytosis in the Systemic Delivery of FBP to the Brain 

**Figure 3:**
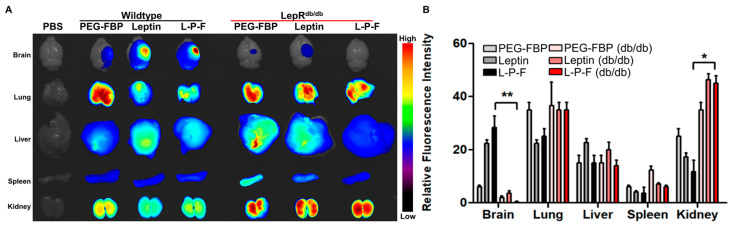
Leptin–leptin receptor binding facilitates Leptin-PEG-FBP delivery to the brain. Wildtype and leptin receptor-deficient (db/db) mice were subjected to photothrombotic stroke, followed by administration of the indicated peptides (2.5 mg/kg Alexa^647^-labelled peptides, i.v.). Organs were collected at 12 h post-delivery and imaged on Kodak imaging station. (**A**,**B**) Representative images and quantification of fluorescence intensities after necropsy. Control mice were inoculated with PBS. Fluorescence intensities measured in arbitrary pixel values were normalized to fluorescence intensities in the PBS-treated group. The data represent mean ± SD (*n* = 5 per group). The data in (**B**) were analyzed using Mann–Whitney U test (* *p* < 0.05; ** *p* < 0.01).

To confirm the specificity of leptin-mediated FBP delivery to the brain, we systemically administered Alexa^647^-conjugated leptin alone, PEG-FBP, and leptin-PEG-FBP in leptin receptor-deficient db/db mice subjected to photothrombosis (Figure 3A). The imaging of organs and quantification of fluorescence intensity from the isolated organs 12 h after inoculation revealed no FBP deposition in the brains of receptor-deficient db/db mice, despite strong localization in the wildtype mice (Figure 3A). Thus, leptin peptide proved to be ineffective in delivering FBP, specifically in the form of leptin-PEG-FBP, to the brains of leptin receptor-deficient db/db mice. We did not observe a difference in fluorescence intensity in other peripheral organs, except for the kidneys, where db/db mice exhibited a high intensity, suggesting the drainage of the peptide from the body (Figure 3B). Our results confirm that FBP is delivered to the brain through the leptin/leptin receptor interaction.

### 2.4. Localization of Intravenously Delivered Leptin-PEG-FBP in Infarcted Regions of MCAO Rat Models with Ischemia

**Figure 4:**
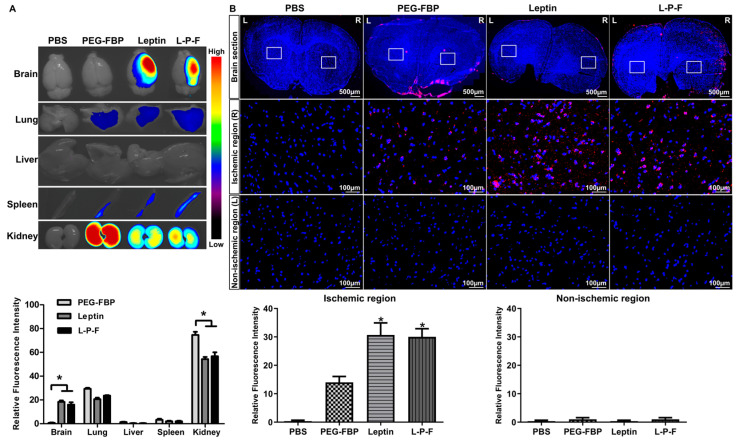
Systemic delivery of leptin-PEG-FBP targets the ischemic region in the brain of MCAO rat model. Rats underwent middle cerebral artery occlusion, followed by the administration of the indicated Alexa^647^-labelled peptides (2.5 mg/kg, i.v.). Organs were harvested 24 h post-inoculation and imaged on Kodak imaging station. (**A**) Representative images and quantification of fluorescence intensities after necropsy. Control mice were inoculated with PBS. Fluorescence intensities measured in arbitrary pixel values were normalized to the intensities of the PBS-treated group. (**B**) Representative confocal microscopy images (lower panel) from brain sections (upper panel) show localization of Alexa^647^-labelled peptides (red) and DAPI-stained nuclei (blue) in MCAO brain tissues. FBP was exclusively labelled in both PEG-FBP and leptin-PEG-FBP. Data were analyzed using Mann–Whitney U test. Data represent the mean ± SD (* *p* < 0.01).

To explore the feasibility of ischemic-specific FBP delivery, we administered Alexa^647^-conjugated peptides to a rat middle cerebral artery occlusion (MCAO) ischemic stroke model. The peptides were delivered via the intravenous route (2.5 mg/kg) and the brain and peripheral organs were examined 24 h post-inoculation. Consistent with our findings in mouse models, we observed a significant localization of leptin and leptin-PEG-FBP in the brain. There was no detectable presence of PEG-FBP in the brain, highlighting the crucial role of leptin in facilitating brain delivery (Figure 4A). Interestingly, we observed a significant fluorescence intensity exclusively in the ischemic region of the brain treated with leptin-PEG-FBP, contrasting with the more widespread fluorescence spectrum in the leptin-treated group. Notably, the rats treated with PEG-FBP did not exhibit brain delivery, further substantiating the role of leptin in successful delivery to the brain. Next, we conducted an in-depth analysis to validate the localization through confocal microscopy. The microscopy data further confirmed the presence of strong Alexa^647^ positive cells in both the leptin- and leptin-PEG-FBP-administered MCAO ischemic model (Figure 4B). Consistent with our findings from organ imaging data, we observed strong Alexa^647^-positive cells in the ischemic region, with no detectable signals in the non-ischemic region (Figure 4B). Our data strongly support the leptin-mediated transcytosis of FBP to the brain in the rat ischemic model.

### 2.5. The Systemic Delivery of Leptin-PEG-FBP Alleviates Brain Cell Death in Rat Ischemic Brain Models

**Figure 5:**
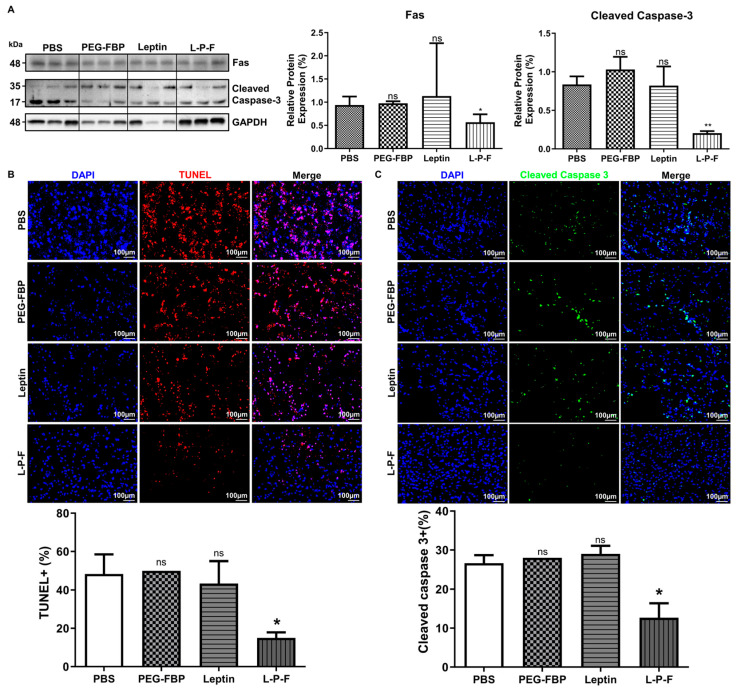
Leptin-PEG-FBP attenuates apoptosis in the MCAO rat model. MCAO rats were systemically treated with the indicated peptides (5 mg/kg, i.v.) at 48 h post-MCAO. (**A**) Representative Western blots and quantification of protein expression for Fas and cleaved caspase-3 in the brain of MCAO mice at 48 h post-MCAO. Protein expression was normalized to GAPDH using Image J software version 1.54d. (**B**) Representative microscopy images of TUNEL-stained brain sections (red) and quantification of TUNEL-positive cells in the ischemic region of MCAO after necropsy. (**C**) Representative microscopy images of cleaved caspase-3-stained brain sections (green) and quantification of positive cells in the ischemic region of MCAO after necropsy. DAPI was used to stain nuclei (blue). Data represent mean ± SD (*n* = 3 animals per group) and were analyzed using Mann–Whitney U test. Data represent the mean ± SD (* *p* < 0.01; ** *p* < 0.01; ns: not-significant).

Ischemic stroke conditions in rat MCAO models lead to brain damage primarily through apoptosis [24]. To assess the therapeutic effects of leptin-PEG-FBP on Fas-mediated apoptosis, we administrated 5 mg/kg of FBP systemically at 3 h and 6 h post-MCAO. At 48 h post-MCAO, we conducted necropsy to estimate the protein levels of Fas and cleaved caspase-3 in the brain samples. Ischemic stroke induced an elevated level of Fas and cleaved caspase-3, similar to the PBS-treated MCAO rats. However, the leptin-PEG-FBP treatment resulted in a 50% reduction in Fas expression and an 80% reduction in downstream cleaved caspase-3 expression compared to that of PEG-FBP and that of leptin-treated MCAO rats (Figure 5A). Furthermore, confocal microscopy data revealed higher levels of TUNEL-positive cells in the ischemic region at 48 h post-MCAO. Approximately 52% of cells were TUNEL-positive in the PBS-treated group, 55% in the PEG-FBP, and 47% in the leptin-treated group (Figure 5B). However, leptin-PEG-FBP treatment significantly reduced the number of TUNEL-positive cells to 18%. Consistent with Western blot data, the immunohistochemistry of the brain demonstrated an elevated expression of cleaved caspase-3 in the ischemic region. Approximately 26% of cells were caspase-3 positive in the PBS-treated group and these levels were reduced by 12% in the leptin-PEG-FBP-treated group (Figure 5C). As expected, there were no significant changes in the TUNEL assay of cleaved caspase-3 positive cells in the leptin- and PEG-FBP-treated MCAO rats. Our data demonstrate that leptin-PEG-FBP can lower the activity of Fas and downstream signaling, thereby reducing apoptosis in the MCAO rat model.

### 2.6. Reduction of Ischemic Infarction in the Rat MCAO with Systemic Leptin-PEG-FBP Treatment 

**Figure 6:**
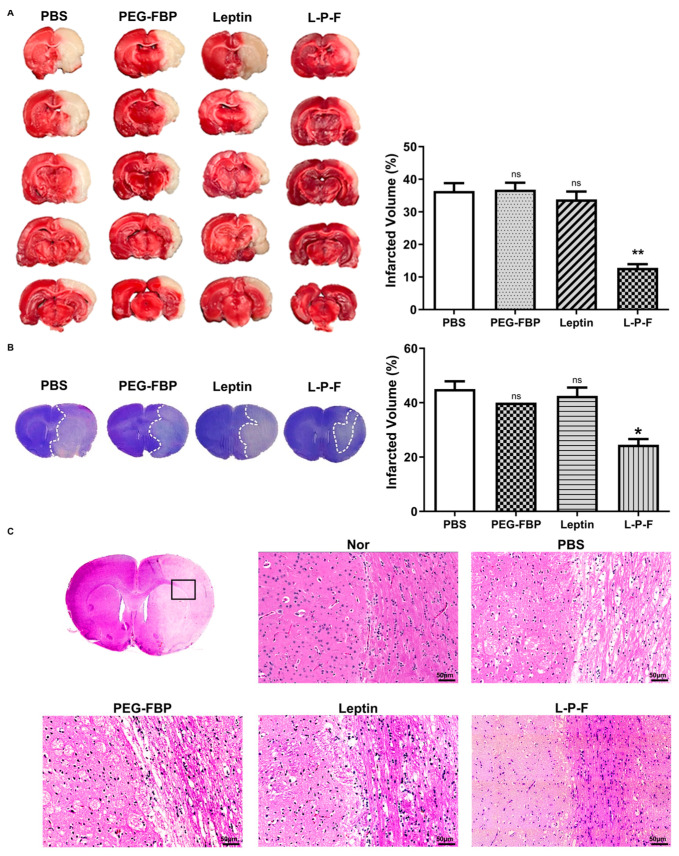
Improvement of MCAO-induced infarction and histopathological changes with leptin-PEG-FBP treatment. MCAO rats were systemically treated with indicated peptides (5 mg/kg, i.v.) at 48 h post-MCAO. (**A**) Brain slices stained with TTC after necropsy. Quantitative analysis of infarction is shown on the right. Data represent mean ± SD (*n* = 5 animals per group). (**B**) Images of Nissl-stained brain slices (left) and quantitative analysis of infarction volume on the right. (**C**) Representative images of hematoxylin and eosin (H&E) brain sections from normal or MCAO-subjected animals after treatment. Data were analyzed using Mann–Whitney U test. Data represent the mean ± SD (* *p* < 0.01; ** *p* < 0.01; ns: not-significant).

The induction of apoptosis in the penumbra after MCAO results in brain damage, leading to the formation of infarcts or areas of tissue damage [9]. To examine the impact of leptin-PEG-FBP on brain infraction, we conducted triphenyltetrazolium chloride (TTC) staining at 48 h post-treatment. The infarct size significantly expanded to almost the entire right hemisphere of the brain within 48 h post-MCAO in the PBS-, PEG-FBP-, and leptin-treated groups, with an infarction volume of 37%, 35%, and 32%, respectively (Figure 6A). However, the leptin-PEG-FBP treatment significantly reduced the infarct area by approximately 70% compared to that of the PBS treatment. Additionally, Nissl-stained brain slices revealed substantial apoptosis in neuronal cells on the right side of brain slice in PBS-, PEG-FBP-, and leptin-treated rats (Figure 6B) compared to that of the rats treated with leptin-PEG-FBP. The leptin-PEG-FBP-treated group exhibited approximately a 20% infarction volume, while rats treated with PBS, PEG-FBP, or leptin consistently showed around a 45% infarction volume (Figure 6B). Both TTC and Nissl-staining data indicated that approximately 50% of brain cells were recovered in the leptin-PEG-FBP-inoculated group. Hematoxylin and eosin (H&E) staining of brain sections also revealed significant damage to the tissue morphology in the right hemisphere of the brain in PBS-, PEG-FBP-, and leptin-treated rats (Figure 6C). In contrast, tissue damage was reduced and highly attenuated in the leptin-PEG-FBP-inoculated group. Our data demonstrate that the leptin-PEG-FBP treatment can rescue brain cells from brain damage.

## 3. Discussion

While there have been substantial advancements in improving drug delivery to specific organs, achieving targeted delivery to the brain remains a considerable challenge, primarily due to the presence of the blood–brain barrier (BBB). A clinically viable approach involves harnessing endocytic ligand receptors on the endothelial cells of the brain for drug delivery [55]. In a previous study, a 30 amino acid leptin-derived peptide demonstrated specific localization in the brain by binding to its corresponding receptor expressed throughout all regions of the brain [48]. Although this ligand-based approach facilitates binding to receptors on brain endothelial cells, ensuring effective drug delivery to the brain, it is limited in its circulation capability. To overcome this limitation, the use of linkers between carriers and therapeutic drugs is often necessary for efficient delivery to the target region in the brain. Hence, in this study, we opted for the use of PEG-2K as a linker. 

In this study, we aimed to evaluate the therapeutic potential of systemically delivering FBP to inhibit the Fas–FasL interaction, thereby reducing neuronal cell death in ischemic stroke. We first examined whether leptin-PEG-FBP could inhibit Fas-mediated apoptosis and found that the conjugated peptide retained the anti-apoptotic effects of FBP. Next, we observed that the intravenous delivery of leptin-PEG-FBP localized to Fas-expressing brain regions in photothrombotic stroke mouse models of brain ischemia. Our findings highlight the importance of PEG in facilitating the transport of the leptin-PEG-FBP peptide through the cerebrospinal fluid to the infarction area, as supported by the biodistribution data (Figure 2C). At the clinical level, PEG polymers are FDA-approved excipients widely used in topical, oral, and intravascular formulations [56]. The metabolism of PEG is mediated by alcohol dehydrogenase, aldehyde dehydrogenase, CYPs, and sulfotransferase [56,57,58]. Furthermore, PEG polymers undergo biodegradation either through the renal or hepatobiliary pathway, depending on their molecular weights. PEG polymers below 20 kDa are cleared through renal filtration, whereas larger PEG polymers are primarily eliminated through liver sequestration followed by biliary excretion [56,58,59]. We observed that the efficiency of delivery of leptin-FBP to ischemic regions was lower when not conjugated through PEG, underscoring the importance of PEG conjugation to FBP in the effective targeting of the ischemic brain region (Figure 2B). Furthermore, prior studies have also shown that the leptin peptide, when administered alone, targets only the hippocampus [48]. However, our findings show the specific localization of leptin-PEG-FBP to the infarction region where Fas is expressed (Figure 3 and Figure S2). Our findings have implications for the importance of PEG in bioconjugation, drug delivery, and in enhancing in vivo stability and solubility, as previously reported [60,61,62]. 

Additionally, we observed an increased expression of Fas in the mouse model of ischemia induced using photothrombosis. Importantly, the fluorescence-labeled leptin-PEG-FBP exhibited successful delivery specifically to the infarcted region of the ischemic brains. This localization was absent in wildtype mice, emphasizing the need for Fas expression to deliver FBP and highlighting the effectiveness of leptin-mediated brain delivery. To support the specificity of leptin-mediated delivery to the brain, our experiments in leptin receptor-deficient mice (db/db) confirmed that fluorescence-labeled leptin, PEG-FBP, and leptin-PEG-FBP peptides did not bind to the brain in a photothrombotic stroke model (Figure 3). The absence of binding from mice treated with leptin, PEG-FBP, or leptin-PEG-FBP in leptin receptor-deficient mice underscores the importance of the leptin/leptin receptor interaction in our novel delivery process.

The intravenous delivery of leptin-PEG-FBP in the MCAO rat model demonstrated localization in infarcted brain regions, surpassing the effectiveness of PEG-FBP. The observed deposition of leptin-PEG-FBP in the ischemic areas aligns with therapeutic potential as it reduces apoptosis, as evidenced by the decreased Fas and cleaved caspase-3. In addition to delivery, our study investigated the therapeutic impact of leptin-PEG-FBP on brain cell death in ischemic stroke. The reduction in apoptosis was further supported by the attenuation of the infarcted volume, improved histology, and reduced tissue damage in the brain, aligning with observations from previous studies where FBP was intranasally delivered to treat brain ischemia [30]. 

Our study is not without limitations. In our animal model, we adapted the MCAO rat model and the photothrombotic mouse model, which are commonly employed experimental models for investigating ischemic stroke [63]. The MCAO rat model has several advantages, including high reproducibility, highly controllable reperfusion, and no necessity for a craniectomy. The MCAO rat model also closely mimics human strokes by occluding the middle cerebral artery and subsequently exhibiting a penumbra [64]. Despite its widespread use and characterization, inherent species differences limit the direct translatability of the MCAO rat model to human stroke [65]. The anesthesia and surgical procedures involved in MCAO may impact the response and recovery differently compared to human strokes, leading to potential differences in observed effects [66]. MCAO is also unsuitable for thrombolysis studies and is associated with increased hemorrhage when certain suture types are used [63,67]. Variability in the extent and location of the occlusion, influenced by the experimenter’s proficiency, can lead to inconsistent results [68]. While offering precise spatial control over the location and extent of the ischemic injury for targeted studies, and despite its high reproducibility compared to some other stroke models, the simplicity of the photothrombotic mouse model’s induction mechanism may limit its ability to represent the full complexity of human stroke [69]. For example, in the rat model, it causes early vasogenic edema, which is atypical in human stroke [63]. Given these limitations, we utilized the photothrombotic mouse model to confirm the reproducibility of systemic delivery of leptin-PEG-FBP and to verify leptin-mediated delivery. The therapeutic efficacy was further validated through the MCAO rat model.

This study not only validates prior research indicating the neuroprotective effects of FasL elimination or suppression of the Fas–FasL system, but also extends these findings by demonstrating the efficacy of leptin-PEG-FBP in inhibiting apoptosis, particularly in Fas-expressing cells within ischemic brain regions. The success of leptin-mediated transcytosis in delivering the FBP peptide underscores its potential as a therapeutic strategy for attenuating brain cell death in the context of ischemic stroke. Our study’s comprehensive findings advocate for the consideration of a combined therapy involving leptin-PEG-FBP and tissue plasminogen activator (tPA), pointing towards enhanced clinical effectiveness in stroke treatment. This combination addresses both fast apoptosis triggered by acute ischemia and slow apoptosis in the penumbra, offering a dual targeted approach to stroke management. The synergistic effects of these agents suggest a promising avenue for future clinical applications, potentially revolutionizing the treatment landscape for ischemic stroke by concurrently targeting distinct apoptotic pathways associated with different stages of the ischemic insult. This research contributes valuable insights into the development of advanced therapeutic modalities for stroke intervention. However, given that there are reports suggesting that leptin itself can alleviate stroke [70], further studies must be conducted to gain a more comprehensive understanding of the role of leptin in ischemic strokes.

## 4. Materials and Methods

### 4.1. Peptides

Peptides were synthesized from Anygen (Gwangju, Korea). Peptides were dissolved in dimethyl sulfoxide (DMSO) at 100 mg/mL. The sequences of peptides are depicted below.

Leptin (YQQVLTSLPSQNVLQIANDLENLRDLLHLLGGGC) [48]
FBP (YCDEHFCY) [51]
Control FBP (YCNSTVCY) [51]
RV-MAT (MNLLRKIVKNRRDEDTQKSSPASAPLDGGGC) [45]

Leptin-PEG-FBP was prepared by dissolving leptin peptide in DMSO at a concentration of 100 mg/mL and then diluted in DPBS (pH 7.4) to 10 mg/mL. To block the N-terminal of the leptin peptide, 99.5% acetic anhydride (Sigma-Aldrich, St. Louis, MO, USA) was added, equivalent to the mass of the leptin peptide. The reaction was stirred for 1 h at 4 °C [71].

Next, we conducted PEGylation with the maleimide conjugation process. The leptin peptide was conjugated with Mal-PEG2000-NH2 (JenKem Technology, Plano, TX, USA) at a 1:1 molar ratio. This reaction occurred overnight at 4 °C in the dark. Subsequently, dialysis using a 2000 Da cassette in DPBS (pH 7.4) was conducted for 2 h at 4 °C [72,73].

The final step involved activating FBP, followed by conjugation through an EDC/sulfo-NHS reaction with leptin. FBP was dissolved in DMSO at a concentration of 100 mg/mL and diluted to 1 mg/mL in 0.1 M MES buffer (pH 6.5). EDC and sulfo-NHS (Thermo Scientific) were equilibrated to room temperature before being added to FBP [74,75]. The FBP activation process occurred with vortexing for 15 min at room temperature. Activated FBP was then conjugated with leptin-PEG for 3 h at room temperature. To purify the coupled peptides, dialysis with a 4000 Da cassette in DPBS (pH 7.4) was performed for 2 h at 4 °C.

### 4.2. Cell Culture Studies

Neuro2a mouse neuroblastoma cells (N2a) were obtained from ATCC (Rockville, MD) and cultured in Dulbecco’s modified Eagle’s medium (DMEM, Gibco) containing 10% fetal bovine serum, penicillin (100 IU/mL), and streptomycin (100 μg/mL). To mimic the in vitro ischemia/reperfusion environment, N2a cells were maintained in a hypoxic condition (94% N_2_, 5% CO_2_, 1% O_2_, 37 °C) in oxygen glucose deprivation media (OGD, Life Technologies, Carlsbad, CA, USA) for 24 h. The cells were then reoxygenated in DMEM supplemented with 10% FBS (5% CO_2_, 20% O_2_, 37 °C) for another 24 h and cultured in Dulbecco’s modified Eagle’s medium (DMEM) (Gibco) containing high glucose, supplemented with 10% calf serum and 1% penicillin-streptomycin. To analyze the functional effects of leptin-PEG-FBP, normoxia- and hypoxia-induced N2a cells were seeded in a six-well plate at 2 × 10^5^/well with serum-free DMEM and treated with leptin-PEG-FBP at 1 mM for 6 h followed by exposure to a soluble Fas ligand (5 nM). After 6 h, the cells were stained with Annexin V- PE (BD Pharmingen™, Baltimore, MD, USA) according to the manufacturer’s instructions [30].

### 4.3. Animals

All experiments were performed in compliance with guidelines and using protocols approved by Hanyang University’s Institutional Animal Care and Use Committee (IACUC). Three different animals were studied: male SD rats (*n* = 5, 9 weeks), male BALB/c mouse (*n* = 5, 7 weeks), and male Leprdb/db mice (*n* = 5, 6 weeks).

### 4.4. Experimental Stroke Models

Acute cerebral ischemia was produced using a 1 h occlusion of the right middle cerebral artery (MCAO) in Sprague–Dawley rats weighing 280–320 g (Orient Bio, Seoul, Republic of Korea), as previously described [76]. Briefly, ischemic stroke injury was induced by ligating the external carotid artery (ECA) with a silk thread followed by occlusion of the middle cerebral artery (MCA) through the ECA to the internal carotid artery (ICA) by inserting a nylon suture. Then, the common carotid artery (CCA) was completely occluded using a clip. After 1 h of occlusion, reperfusion was initiated by pulling out the suture.

A photothrombotic stroke to unilaterally lesion the sensorimotor cortex was induced on the right hemisphere. Rose Bengal dye (0.2 mL of 8 g/L) was then injected intraperitoneally. The thinned skull region was then illuminated with a 0.02 mW green laser for 10 min, the scalp was sutured, and the animal was allowed to recover.

### 4.5. Bio-Distribution

For bio-distribution studies, Alexa^647^-conjugated peptides were intravenously (IV) administered at 2.5 mg/kg. The peptides were complexed with Alexa^647^ dye (1:20 weight ratio) and then intravenously injected into mice at 3 h post-MCAO. Fluorescence was analyzed at designated time points. After 12 h inoculation, fluorescence was measured in the brain, lung, liver, spleen, and kidney using image station (Kodak, Stamford, CT, USA).

### 4.6. Real-Time RT-PCR

Total mRNA was isolated using the RNAiso plus (Takara). cDNA was prepared by using One-Step RT-PCR Series (BIOFACT™) with 5 μg of total RNA. The quantitative evaluation of mRNA was conducted with a 7500 Real-Time PCR system using SYBR premix ExTaq perfect real time (Takara, Shiga, Japan). The data were analyzed using the ΔΔCt method and normalized to GAPDH mRNA levels. All primer sequences used are depicted below.

Fas (CD95) forward 5′-GGAGGTGGTGATAGCCGGTAT-3′ reverse 5′-TGGGTAATCCATAGAGCCCAG -3′
GAPDH forward 5′-AGGTCGGTGTGAACGGATTTG-3′, reverse 5′-TGTAGACCATGTAGTTGAGGTCA-3′

### 4.7. TTC Staining

Pathology in brain tissues was analyzed by staining 2 mm brain slices in 2% 2,3,5-tryphenyltetrazolium chloride (TTC, Sigma Aldrich, St. Louis, MO, USA) for 15 min at 37 °C. The infarcted volume was calculated after fixation using Image J software version 1.54d, as described [30,76].

### 4.8. Nissl Staining

Infarcted brain volume was analyzed using Nissl staining. The OCT-embedded brain samples for cryosection were fixed using 4% PFA at room temperature and subjected to 0.1% crystal violet solution following standard protocol [77]. The stained sections were cover-slipped and blindly pictured using full-screening scanner. The infarcted volume was calculated using Image J software version 1.54d.

### 4.9. Histology

Rats were sacrificed and perfused with PBS. Brains were carefully removed, embedded in OCT compound (Sakura Finetek, Alphen aan den Rijn, The Netherlands), rapidly cryopreserved in frigid isopentane chilled with liquid nitrogen, and stored at −80 °C until the subsequent sectioning procedure [78]. Before the sectioning process, all materials and samples were placed into a cryostat set at −20 °C and allowed to stabilize for a minimum of 15 min. Subsequently, 10 µm lesion-centered coronal cryo-sectioning was conducted, and the sections were stained with hematoxylin and eosin (H&E) for morphological analysis.

### 4.10. TUNEL Assay

Terminal deoxynucleotidyl transferase dUTP nick end labeling (TUNEL) assay was performed to determine apoptosis in the brain tissue infarct area using an apoptosis detection kit (Roche Applied Sciences, Penzberg, Germany). The assay was conducted according to the manufacturer’s instructions [77]. Briefly, all samples were cut using cryo-section and fixed with 4% PFA for 15 min at room temperature. The tissue sections were permeabilized and incubated with the TUNEL reaction mixture including TdT and fluorescence-labeled dUTP at room temperature. Nuclei were stained with DAPI. The apoptotic cells were analyzed with a confocal microscope. TUNEL-positive cells were counted using Image J software version 1.54d.

### 4.11. Immunohistochemistry

Tissue samples from the brains were cut using cryo-sectioning and fixed in 4% PFA for 1–2 days at 4 °C for further experiments. In brief, the sections were blocked with 1% BSA and stained with a rabbit anti-cleaved caspase-3 antibody (1:100, Abacm, Waltham, MA, USA) in phosphate-buffered saline (PBS) supplemented with 0.3% Triton X-100 at 4 °C overnight under humid conditions [79]. Subsequently, the sections were incubated with an FITC-labeled goat anti-rabbit secondary antibody (1:500, Abacm, ab97050) for 2 h at room temperature in the dark. In all cases, samples were counterstained with DAPI and FITC signal was detected using confocal microscopy. Fluorescent positive cells were counted using Image J software version 1.54d.

### 4.12. Western Blot

Western blotting of brain tissue was performed with 30 μg of protein transferred onto PVDF transfer membrane from a 10% SDS-PAGE. The blots were probed with rabbit anti- cleaved caspase-3 (1:1000, Abacm, ab52293) and rabbit polyclonal anti-Fas (1:1000, Abcam, ab82419). Secondary polyclonal antibodies were also probed to rabbit IgG coupled to HRP (1:2000, Abcam, ab97051). The blots were developed using ECL Western blotting substrate (Promega, Medison, WI, USA).

### 4.13. Statistical Analysis

The data are expressed as the mean ± SEM or SD, as appropriately indicated in specific instances. Significance in mean values between two groups was evaluated using the Mann–Whitney U test, while differences among more than two groups were analyzed using one-way ANOVA using Graphpad Prism 7 software. A significance level of *p* < 0.05 was considered statistically significant.

## 5. Conclusions

Our study demonstrates the inhibition of the Fas–FasL interaction by systemically delivering a leptin-PEG-Fas-blocking peptide to brain regions affected by ischemic stroke. This study underscores the potential of precise molecular interventions in treating ischemic stroke and introduces a novel approach by utilizing a leptin-derived peptide for the targeted brain delivery of FBP. The successful inhibition of Fas-mediated apoptosis and subsequent recovery in animal models suggest a promising avenue for further exploration of this fusion peptide as a therapeutic strategy in ischemic stroke management.

## Data Availability

All data are present within the manuscript and Supplementary Materials.

## Supplementary Materials

The following supporting information can be downloaded at: https://www.mdpi.com/article/10.3390/ijms25010661/s1.
