# Supplementary material for: Systemic Treatment with Fas-Blocking Peptide Attenuates Apoptosis in Brain Ischemia
Source: Int J Mol Sci. 2024 Jan 4;25(1):661. doi: 10.3390/ijms25010661 (PMC10780202; doi:10.3390/ijms25010661)
Supplement: Supplementary file 1 [file ijms-25-00661-s001.zip › ijms-2800729-supplementary.pdf]

Supplementary Materials

Systemic treatment of Fas-blocking peptide attenuates apoptosis in brain ischemia

Sungeun Chung[1,†], Yujong Yi[1,†], Irfan Ullah[1,2], Kunho Chung[1,3], Seongjun Park[1], Jaeyeoung Lim[1], Chaeyeon Kim[1], Seon-Hong Pyun[1], Minkyung Kim[1], Dokyoung Kim[4], Minhyung Lee[1], Taiyoun Rhim[1,*] and Sang-Kyung Lee[1,*]

Total Supplementary Figures (S1~S2)

Figure S1. Effect of PEGylation length on apoptosis inhibition by FBP peptides

Figure S2. Leptin peptide-mediated brain delivery in rats

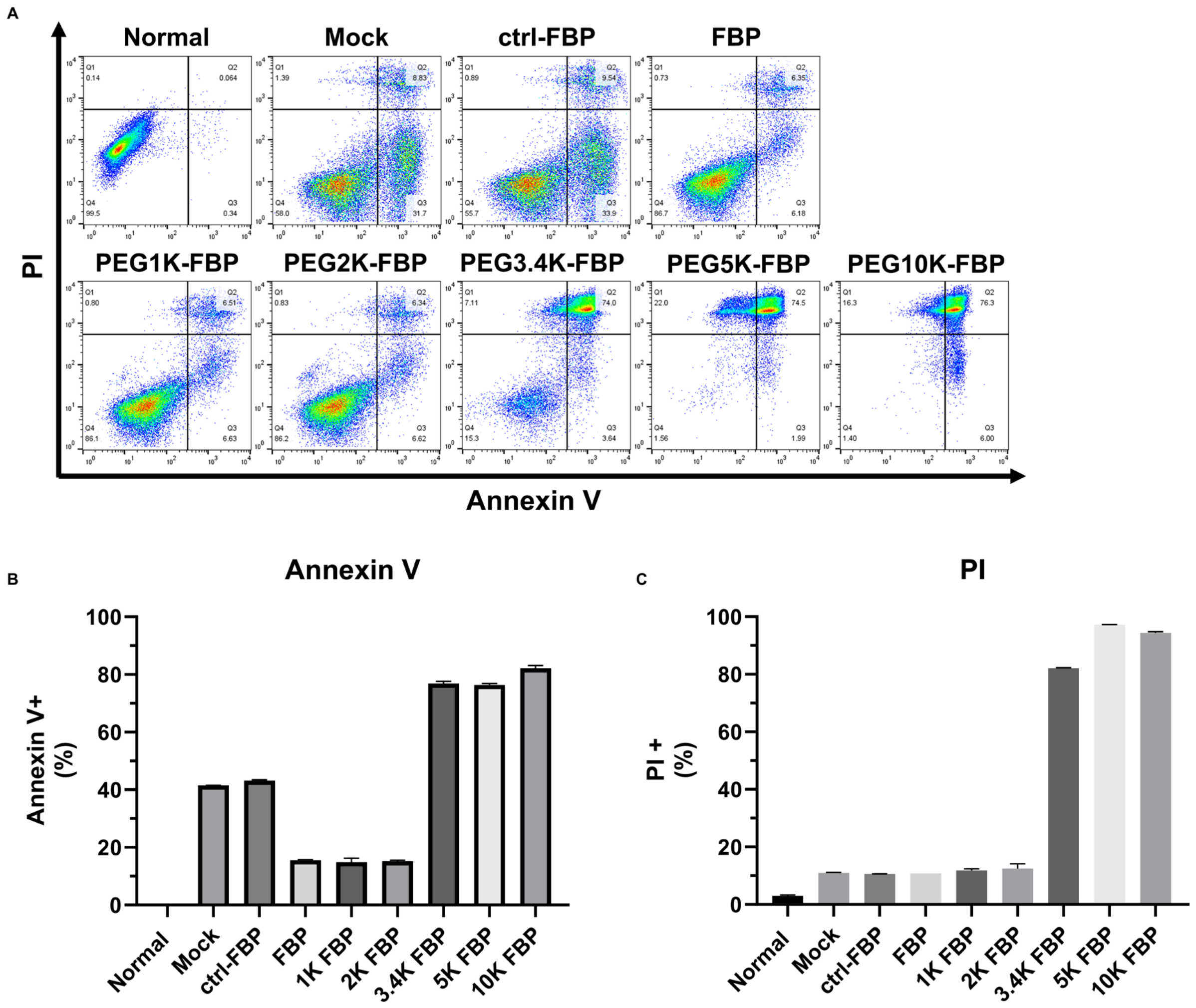

Figure S1. Effect of PEGylation length on apoptosis inhibition by FBP peptides. (A) Apoptosis in Jurkat cells was assessed 3 hr post-treatment with membrane-bound soluble FasL (20 ng/ml), with and without peptides at a concentration of 300 μM using Annexin V staining. **(B-C)** Bar graphs illustrating the percentage of cells in early (B) and late (C) apoptosis. The comparison includes non-treated, ctrl-FBP, FBP, and various PEGylated FBP (1K, 2K, 3.4K, 5K, 10K) after FasL treatment in Jurkat cells. Data represent averages ± SD from three independent experiments (*$p < 0.05$).

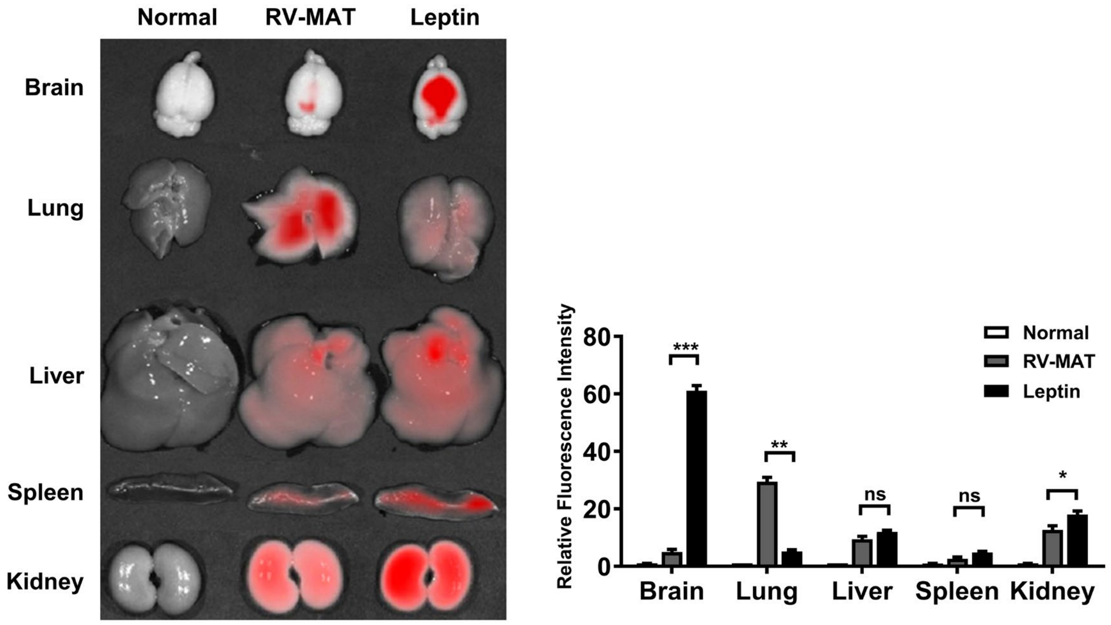

Figure S2. Leptin peptide-mediated brain delivery in rats. Biodistribution (left panel) of A647-conjugated peptides delivered intravenous (n=3) in normal rats. Brain, lung, liver, spleen and kidney were examined 24 hr post-inoculation. Rats were treated with RV-MAT, the control peptide and the leptin peptide. Cumulative data (right panel) show relative fluorescence intensities measured in arbitrary pixel values for examined tissues. The measurements are compared to the normal group and are based on three independent animals.
